# Are we there yet? Shared Decision-Making Needs in Older Adults with Atrial Fibrillation and Multimorbidity

**DOI:** 10.1093/geroni/igaf122.3156

**Published:** 2025-12-31

**Authors:** Hawa Abu, Jane Saczynski, Michelle Nabi, Annie Ferris, Jerry Gurwitz, David Dosa, Alok Kapoor, David McManus

**Affiliations:** University of Massachusetts Chan Medical School, Worcester, Massachusetts, United States; Northeastern University, Boston, Massachusetts, United States; University of Massachusetts Chan Medical School, Worcester, Massachusetts, United States; University of Massachusetts Chan Medical School, Worcester, Massachusetts, United States; University of Massachusetts Chan Medical School, Worcester, Massachusetts, United States; University of Massachusetts Chan Medical School, Worcester, Massachusetts, United States; University of Massachusetts Chan Medical School, Worcester, Massachusetts, United States; University of Massachusetts Chan Medical School, Worcester, Massachusetts, United States

## Abstract

**Background:**

Older adults with atrial fibrillation (AF) and multimorbidity face complex stroke prevention decisions. Identifying those with unmet Shared Decision-Making (SDM) needs can enhance patient-provider communication.

**Objective:**

Examine the prevalence and characteristics of older adults with AF and multimorbidity expressing a need for greater SDM involvement for stroke prevention.

**Methods:**

Patients aged 65 years and older with AF were enrolled in a prospective cohort study (2016-2018) from clinics in Massachusetts and Georgia. Participants with one or more chronic conditions were included. At one-year follow-up, participants on anticoagulation were asked about their preference for greater involvement in decision-making regarding the decision to use anticoagulation and the choice of anticoagulant. Affirmative responses indicated a need for greater SDM engagement. Multivariable logistic regression identified patient characteristics associated with preference for more SDM engagement.

**Results:**

Participants’ (n = 532) mean age was 75 years; 48% were women, and 87% were White. Overall, 41% had 1–4 chronic conditions, 40% had 5–7, and 19% had 8 or more. One-third expressed a need for greater SDM in both anticoagulation initiation and selection of anticoagulant. After multivariable adjustment, participants who were younger (aged 65–74 years), non-White, had lower education level, fewer comorbidities or a higher perceived burden from anticoagulation use, reported a need for greater SDM engagement for stroke prevention.

**Conclusions:**

Clinicians need to recognize the specific needs of older patients with AF and multimorbidity that seek greater SDM involvement for stroke prevention. Providing tailored interventions can enhance the decision-making process for stroke prevention in this vulnerable population.

